# Investigating the mechanism and prognosis of patients with disorders of consciousness on the basis of brain networks between the thalamus and whole-brain

**DOI:** 10.3389/fneur.2022.990686

**Published:** 2022-09-27

**Authors:** Jun Zhang, Hongying Zhang, Fuli Yan, Hengzhu Zhang, Enpeng Zhang, Xingdong Wang, Min Wei, Yunlong Pei, Zhijie Yang, Yuping Li, Lun Dong, Xiaodong Wang

**Affiliations:** ^1^Department of Neurosurgery, Huashan Hospital, Shanghai Medical College, Fudan University, Shanghai, China; ^2^Department of Neurosurgery, Clinical Medical College of Yangzhou University, Yangzhou, China; ^3^Department of Radiology, Clinical Medical College of Yangzhou University, Yangzhou, China

**Keywords:** disorders of consciousness, severe traumatic brain injury, thalamus, brain networks, resting state functional magnetic resonance (rs-fMRI)

## Abstract

**Purpose:**

This study aimed to investigate the changes in the functional connectivity between the bilateral thalamus and the whole-brain in patients with severe traumatic brain injury (sTBI) patients suffering from disorders of consciousness (DOC) and to explore their potential prognostic representation capacity.

**Methods:**

The sTBI patients suffering from DOC and healthy controls underwent functional magnetic resonance imaging. We defined patients with the Extended Glasgow Outcome Score (GOS-E) ≥ 3 as the wake group and GOS-E = 2 as the coma group. The differences in functional connectivity between sTBI and healthy controls and between wake and coma groups were compared. Based on the brain regions with altered functional connectivity between wake and coma groups, they were divided into 26 regions of interest. Based on the *Z*-values of regions of interest, the receiver operating characteristic analysis was conducted to classify the prognosis of patients.

**Results:**

A total of 28 patients and 15 healthy controls were finally included. Patients who had DOC indicated a significant disruption of functional connectivity between the bilateral thalamus and the whole-brain (FDR corrected, *P* < 0.0007). The functional connectivity strength (bilateral thalamus to whole-brain) was significantly different between coma patients who went on to wake and those who were eventually non-awake at 6 months after sTBI (Alphasim corrected, *P* < 0.05). Furthermore, the 26 regions of interest had a similar or even better prognostic distinction ability than the admission Glasgow coma score.

**Conclusion:**

The thalamus-based system of consciousness of sTBI patients suffering from DOC is disrupted. There are differences in the thalamus-to-whole-brain network between wake and coma groups and these differences have potential prognostic characterization capability.

## Introduction

Severe traumatic brain injury (sTBI), affecting millions of people each year, is a serious global public health problem (1, 2). sTBI is a leading cause of death, especially among young people (3). For survivors, however, disorders of consciousness (DOC) is a major challenge (4). Currently, there is no consensus on why sTBI causes DOC (5). Knowing the structure and composition of the normal consciousness networks is the basis and premise for the study of DOC caused by sTBI.

Consciousness is an interesting but controversial scientific issue. From a neuroscience perspective, consciousness originates from the brain, which is a kind of information processing based on the hardware of the brain and is regulated by the consciousness control system (6). From an anatomical point of view, similar to other cortical-subcortical systems, such as the sensory, motor, or limbic systems, the brain networks dedicated to regulating the level of consciousness could be referred to as the “consciousness system” (7). The cortical regions of the consciousness system included medial (medial frontal, anterior cingulate, posterior cingulate, precuneus, and retrosplenial cortex) and lateral (lateral frontal, anterior insula, orbital frontal, and lateral temporal-parietal association cortex) brain surface (6). In addition, the major subcortical components of the consciousness system include the midbrain, superior pons, thalamus, hypothalamus, and basal forebrain (6). In the consciousness system as a whole, the higher-order cortex interacts with the subcortical arousal system (*via* a series of parallel systems), exerting regulation and control on the overall level of consciousness, arousal, and attention (6).

Once the consciousness system is disconnected or the balance is disturbed, a DOC can occur. The advent of resting-state functional magnetic resonance imaging (fMRI) sheds light on DOC exploration. Several studies investigated the features of brain networks in patients with DOC (8–11). Although there is significant methodological heterogeneity among the studies, the study content is based on the consciousness system. Evidence from graph theory indicated that cortical regions in the hubs of the brain networks in health become non-hubs of the brain networks in DOC patients (8). The study by Vanhaudenhuyse et al. (9) analyzed differences in default mode networks in patients with different degrees of DOC. The finding suggested that the functional connectivity strength of the default mode networks was correlated with the level of consciousness (9). In fact, the study indicated that significant impairment of effective connectivity in the fronto-parietal brain network was one of the causes of DOC (10). Whether based on neuroanatomy or functional neural-brain networks theory, scholars agree that the thalamus plays an important role in consciousness (11). Further study revealed that thalamus-related functional connectivity was attenuated in DOC patients (11). The above studies analyzed the DOC mechanism based on multiple disease types (cerebral hemorrhage, brain trauma, cerebral infarction, cerebrovascular disease, etc.), but did not elaborate on a single disease (such as sTBI). The findings of studies may not be fully applicable to traumatic DOC.

In summary, given the bilateral thalamus's crucial role in the generation of consciousness, the purpose of this study was to investigate the changes in the functional connectivity between the bilateral thalamus and the whole-brain in patients with sTBI-type DOC. In addition, most of the studies only explained the DOC-related mechanisms, and the ability of brain networks between the bilateral thalamic and whole-brain to assess patients' wake (6 months after DOC) is unclear, so our study attempts to clarify this.

## Materials and methods

### Participants

The study was a prospective, single-center study. A total of 30 sTBI patients with DOC and 15 health controls were initially included from June 2020 to June 2021.

Inclusion criteria of patients: (1) Age 18–80 years old; (2) the diagnosis of sTBI patients with DOC was confirmed by history, scale—Glasgow coma score (GCS) ≤ 8, and brain imaging data (12, 13); (3) in the 1st to 4th weeks after injury; (4) the work and communication before the sTBI was normal; (5) right-handed; (6) stable spontaneous breathing and vital signs; (7) all guardians agreed to performed this research and signed an informed consent form. Exclusion criteria of patients: (1) Comorbidities other neurological and or psychiatric diseases; (2) non-traumatic DOC; (3) with severe multiple injuries; (4) contraindications for Magnetic resonance imaging (MRI) detection; (5) history of alcohol and drug abuse.

Inclusion criteria of healthy controls: (1) the baseline characteristics were partially matched (age could be moderately biased) with the DOC patients; (2) all controls be informed of the study protocol and signed the consent form; (3) no history of alcohol and drug abuse; (4) no familial neurological and/or psychiatric diseases; (5) no contraindications for MRI detection.

### Prognosis assessment

A professionally trained neurosurgeon was masked to access the neurological prognosis outcomes of DOC patients for 6 months by telephone (close family members were investigated) and/or outpatient follow-up. The Extended Glasgow Outcome Score (GOS-E) was applied to evaluate DOC patients' prognosis (14). We defined patients with GOS-E ≥ 3 as the wake group and GOS-E = 2 as the DOC group (15).

### fMRI data acquisition

The fMRI data of DOC patients (1–4 weeks after sTBI) were collected. The fMRI data were acquired with an 8-channel head coil on a 3.0 Tesla MRI system (Discovery MR750; GE Medical Systems, Milwaukee, WI, USA) located in the Neuroradiology Center. The 8 min fMRI resting-state sequence applied the following parameters: percent phase field view = 100, echo time = 30 ms, repetition time = 2,000 ms, flip angle = 90°, matrix = 64 × 64, slice thickness = 3.5 mm, spacing between slices = 4.2 mm, field of view = 300 × 300 mm, and slices = 33.

Whole-brain three-dimensional structural images were acquired using magnetization-prepared rapid acquisition gradient-echo T1-weighted sequence. The parameters were as follows: echo time = 3.18 ms, repetition time = 8.16 ms, flip angle =12°, matrix = 256 × 256, slice thickness = 1 mm, and slices = 168.

### fMRI data preprocessing

The resting-state fMRI data were preprocessed by Statistical Parametric Mapping 12 (SPM 12) (https://www.fil.ion.ucl.ac.uk/spm/software/). The preprocessing steps included the following processes: (1) Convert Digital Imaging and Communications in Medicine format to Neuroimaging Informatics Technology Initiative format; (2) eliminate the functional data at the first 10 time points; (3) adjust the original position of the structure image to anterior commissure; (4) use the 33rd level layer as the reference layer for slice timing; (5) exclude data with head translation > 2 mm and rotation > 2° and realign head movements; (6) segment the structure image into gray matter, white matter, and cerebrospinal fluid; (7) normalize image to Montreal standard head anatomic space (reslice by 3 × 3 × 3 mm); (8) apply the Gauss kernel of 4 mm with full width and half height for smoothing; (9) delete the linear trend; (10) low-frequency filter 0.01–0.08 Hz.

### fMRI data post-processing

**Figure 1:**
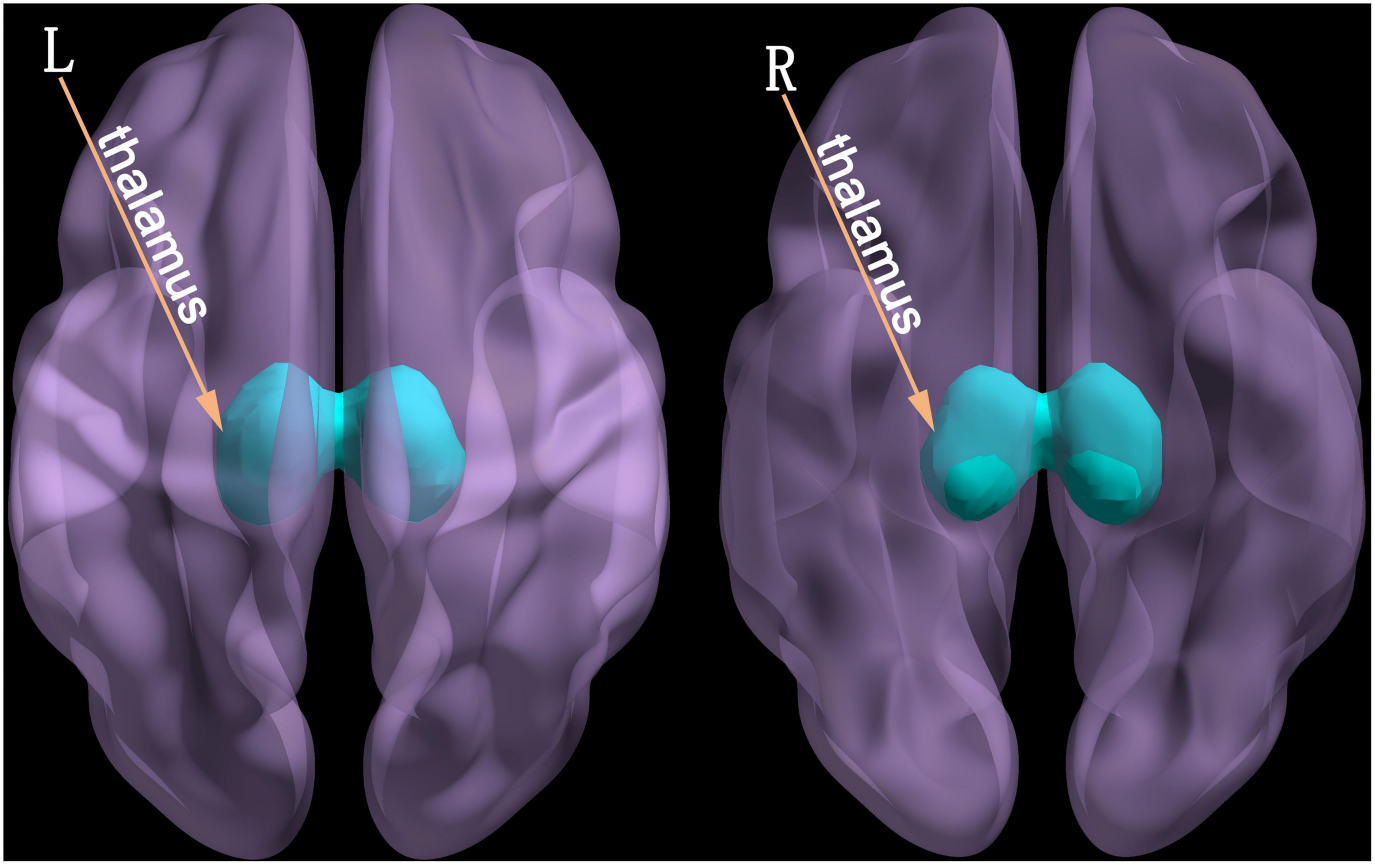
The seed point for functional connectivity analysis - the thalamus (L, left; R, right).

Rest 1.8 (http://www.restfmri.net/forum/REST_V1.8) software was used to post-process the fMRI data. The bilateral thalamus (Figure 1) was extracted as a region of interest based on the automated anatomical labeling (AAL) template (16). Then, the average time series of the voxels in regions of interest were calculated and Pearson's correlation analysis was used on the time series of each voxel in the whole-brain (17). Therefore, the functional connectivity matrix between the bilateral thalamus and the whole-brain voxel was obtained. However, the functional connectivity value was the *R* correlation coefficient (non-normal distribution). Thus, the Fisher's was performed to convert it into a *Z*-value conforming to the normal distribution (18).

A two-sample *t*-test was applied by the Rest 1.8 based on the functional connectivity (DOC patients *vs*. health controls and wake group *vs*. DOC group). The multiple comparison correction was performed using the AlphaSim (clusters > 154, *P* < 0.05) (19, 20) or false discovery rate (FDR) (*P* < 0.05) to correct functional connectivity values and we adjusted for nuisance covariates (cerebrospinal fluid, white matter, head motion coefficient, age, and gender) (19, 21). The areas of different functional connectivity (wake group *vs*. DOC group) were taken as regions of interest. Based on brain regions with altered functional connectivity, 26 regions of interest were manually defined (seed point as the origin and 1.5 mm as the radius). The manual definition process was as follows: (1) The brain regions with altered functional connectivity were identified; (2) Based on xjView (https://www.alivelearn.net/xjview/), allowed the mouse to click the abnormal functional connectivity map one by one; (3) During intensive clicking, and if a different brain region is revealed, it will be recorded as a region of interest. In addition, the AAL template was adopted and aimed to divide the whole brain into 116 regions of interest to examine the robustness of manual segmentation results. The *Z*-values of each region of interest were extracted as variables for prognostic prediction by Rest 1.8.

Pearson's correlation coefficients between each region of interest pair were calculated to form a matrix of *R*-values. Then, similarly, Fisher's was applied to convert the *R*-values matrix into a *Z*-values matrix conforming to the normal distribution (22). A two-sample *t*-test was performed to compare the Z matrix differences between the wake and DOC groups. Differences between groups were presented as a matrix of *P*-values. Furthermore, based on statistical values, a node-side-node brain network was employed to further visualize functional connectivity differences. The nodes represent brain areas, the edges represent interregional functional connectivity, and the diameters of the edges represent functional connection strength.

### Statistics analysis

SPSS 26 (https://www.ibm.com/support/pages/downloading-ibm-spss-statistics-26) and MedCalc (https://www.medcalcsoftware.com/) software were conducted for data analysis. The Kolmogorov-Smirnov was performed to test the normality of measurement data. Normally distributed data were represented by mean ± standard, and comparisons between two groups were by a two-sample *t*-test. Otherwise, the median and its quartile were used, and the rank sum test was applied to compare groups. The Chi-square test was applied to the comparison of count data between groups. Each region of interest and GCS were evaluated for their ability to classify the prognosis using the receiver operating characteristic (ROC) curve. Statistical significance was set at *P* < 0.05.

## Results

### Basic characteristics

A total of 28 DOC patients were finally included, after excluding two patients with significant head movement. Fifteen healthy controls were also included in this study. Baseline characteristics of participants are provided in Table 1. The differences in terms of gender and right-handedness were not statistically significant between DOC patients and healthy controls. However, the difference was statistically significant in age (DOC patients vs. Health controls) (*P* = 0.02). Additionally, the mean value of GCS at admission and GOS-E at 6 months was 5 and 3, respectively, in DOC patients.

**Table 1:** Baseline characteristics of the study subjects.

**Term**	**Healthy controls**	**DOC patients**	**Wake group**	**DOC group**	***P*-value**
Age, (Mean ± SD)	45.07 ± 17.65	58.00 ± 15.39	52.23 ± 17.22	59.38 ± 15.98	0.02^a^; 0.36^b^
Gender, male (%)	10 (66.7)	19 (67.9)	7 (87.5)	8 (61.5)	0.94^a^; 0.20^b^
Right-handed, N (%)	15 (100.0)	28 (100.0)	13 (100.0)	8 (100.0)	-
aGCS, (Mean ± SD)	-	5.00 ± 2.45	5.85 ± 2.38	2.88 ± 1.36	0.002^b^
GOS-E, (Mean ± SD)	-	3.00 ± 1.93	4.69 ± 1.49	2.00 ± 0.00	< 0.001^b^

DOC, disorders of consciousness; aGCS, admission Glasgow coma score; GOS-E, Glasgow outcome score expansion (a. healthy controls vs. DOC patients; b. wake group vs. DOC group).

The patients were divided into wake (*n* = 13) and DOC (*n* = 8) groups according to their GOS-E scores at 6 months. Furthermore, seven patients had died by the final follow-up. Basal demographic characteristics of the two groups (wake and DOC) are shown in Table 1. There were no significant differences between groups in terms of age, gender, and right-handedness. As expected, the differences were statistically significant in admission GCS (*P* = 0.002) and GOS-E at 6 months (*P* < 0.001).

### Differences of connectivity (thalamus to whole-brain) in DOC patients and healthy controls

**Figure 2:**
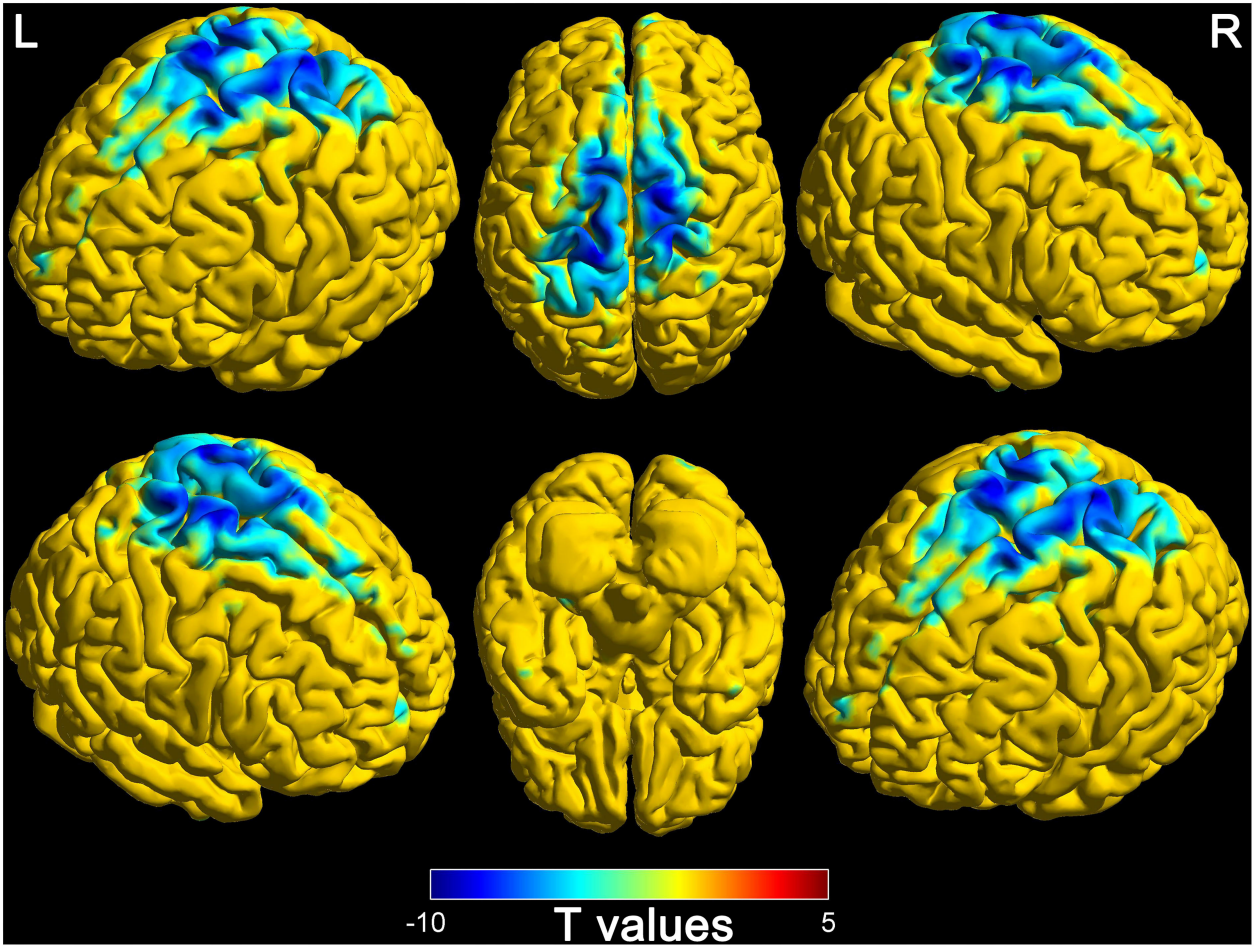
Differences of functional connectivity in thalamus and whole-brain between disorders of consciousness patients and healthy controls (L, left; R, right).

The comparison results between DOC patients and healthy controls are shown in Figure 2. The functional connectivity between the thalamus and some brain regions was decreased in DOC patients, compared with healthy controls (FDR corrected, *P* < 0.0007). The brain regions with weakened functional connectivity were mainly distributed in the core region of the global brain including the frontal lobe, temporal lobe, parietal lobe, occipital lobe, limbic system, cerebellum, and brain stem. The specific brain region features (name, peak point coordinates, statistics *T*-values, and FDR corrected *P*-values) are displayed in Table 2.

**Table 2:** Altered functional connectivity between the thalamus and whole-brain in patients with disorders of consciousness.

**Cluster**	**Regions**	**Peak MNI**	***T*-value**	***P* value**
		**x y z**		
**DOC patients**<**Health controls**				
1	Cerebelum 8	15, −63,−57	−4.27	FDR < 0.0007
2	Cerebellum posterior lobe	−12, −81, −54	−6.25	FDR < 0.0007
3	Left cerebellum, cerebellum inferior	−39, −81, −48	−4.83	FDR < 0.0007
4	Putamen	−30, −9, −51	−5.35	FDR < 0.0007
5	Inferior temporal gyrus	33, −9, −51	−5.36	FDR < 0.0007
6	Cerebellar tonsil, cerebellum anterior lobe	−30, −36, −39	−4.37	FDR < 0.0007
7	Midbrain	6, −30, −9	−4.71	FDR < 0.0007
8	Midbrain, thalamus, sub-thalamic nucleus, substania nigra, red nucleus	−6, −12, −9	−4.17	FDR < 0.0007
9	Middle occipital gyrus, lingual gyrus	27, −102, −9	−5.43	FDR < 0.0007
10	Inferior frontal gyrus, insula, middle frontal gyrus, claustrum	−36, 30, 9	−4.96	FDR < 0.0007
11	Superior temporal gyrus, transverse temporal gyrus, middle temporal gyrus	−42, −30, 6	−4.46	FDR < 0.0007
12	Superior frontal gyrus, medial frontal gyrus	12, 66, 15	−4.83	FDR < 0.0007
13	Inferior parietal lobule, supramarginal gyrus	−48, −42, 15	−4.38	FDR < 0.0007
14	Caudate, lentiform nucleus	−18, −3, 18	−4.67	FDR < 0.0007
15	Precentral gyrus, inferior frontal gyrus	−48, 0, 30	−4.44	FDR < 0.0007
16	Superior frontal gyrus, postcentral gyrus, precentral gyrus, limbic lobe, precuneus, cingulate gyrus, paracentral lobule, middle frontal gyrus, superior parietal lobule, inferior parietal lobule	−15, −39, 78	−10.14	FDR < 0.0007
17	Superior frontal gyrus, medial frontal gyrus	0, 57, 39	−4.22	FDR < 0.0007
18	Precuneus, superior parietal gyrus, superior occipital gyrus	−9, −81, 51	−4.26	FDR < 0.0007

DOC, disorders of consciousness; MNI, Montreal neurological institute.

### Differences of connectivity in thalamus and whole-brain between wake and DOC groups

**Figure 3:**
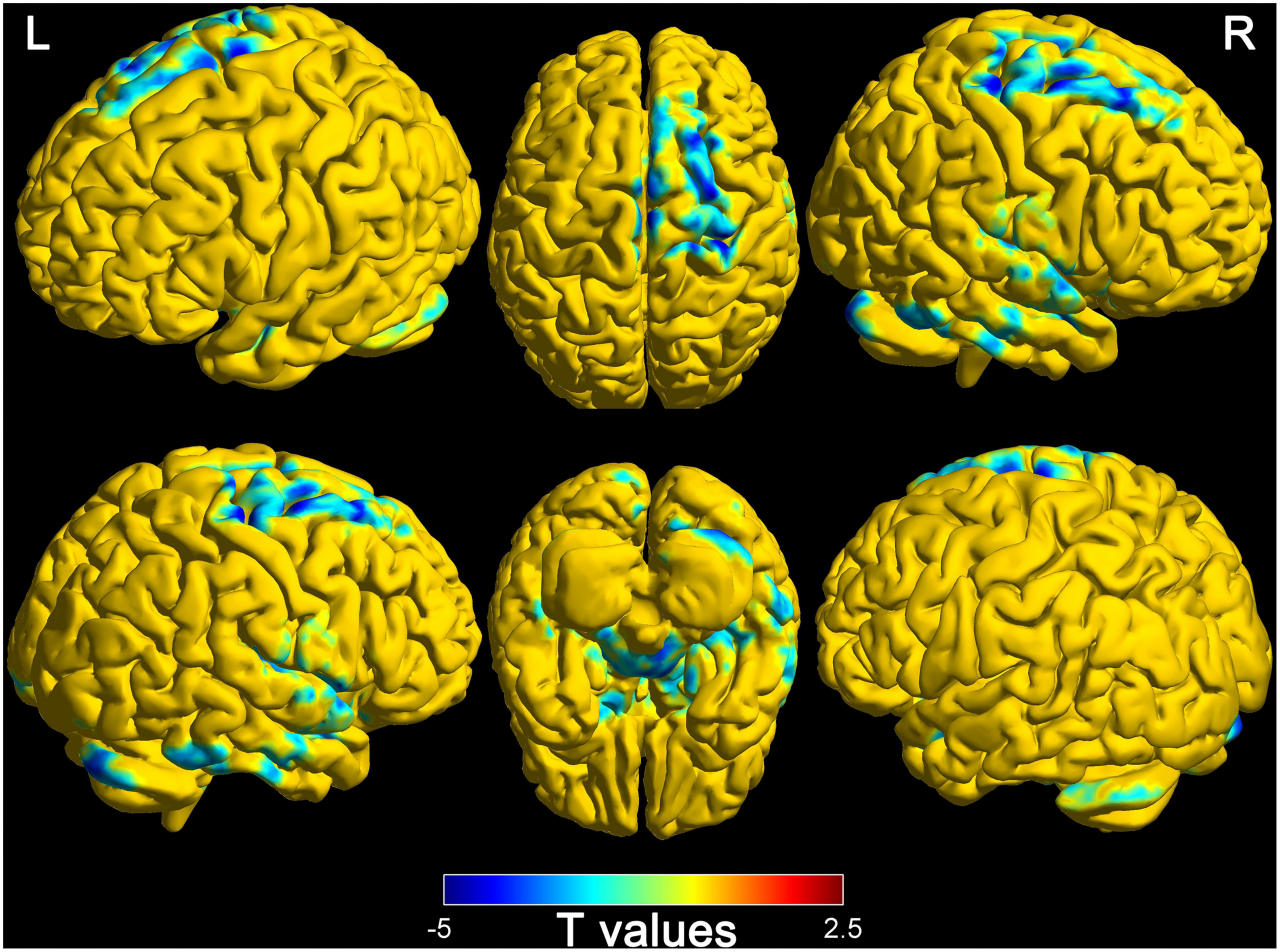
Differences of functional connectivity (thalamus to whole-brain) between wake and DOC groups (L, left; R, right).

Based on the thalamus and whole-brain functional connectivity, there were statistically significant differences between wake and DOC groups (Alphasim corrected, *P* < 0.05). The findings are presented in Figure 3. The brain regions of decreased functional connectivity in the wake group are the frontal lobe, temporal lobe, parietal lobe, occipital lobe, limbic system, cerebellum, and brain stem. Detailed characteristics are provided in Table 3.

**Table 3:** Decreased functional connectivity (thalamus to whole-brain) in wake group.

**Cluster**	**Regions**	**Peak MNI**	**T value**	***P* value**
		**x y z**		
**Wake group**<**DOC group**				
1	Cerebellum posterior lobe, declive, pons, occipital lobe, cerebellum anterior lobe, brainstem, limbic lobe, parahippocampa gyrus, lingual gyrus, fusiform gyrus, inferior temporal gyrus, lentiform nucleus, pallidum, midbrain, hippocampus, cerebellar tonsil, putamen, amygdala, inferior occipital gyrus, insula, inferior frontal gyrus, thalamus, middle temporal gyrus, olfactory cortex, medial frontal gyrus, subthalamic nucleus, cuneus, caudate	39, −87, −24	−5.46	Alphasim < 0.05
2	Extra nuclear, insula, rolandic operculum, superior temporal gyrus, postcentral gyrus, superior temporal gyrus, limbic lobe, transverse temporal gyrus, cingulate gyrus, heschl gyrus, superior temporal gyrus, precentral gyrus, thalamus, middle temporal gyrus, lentiform nucleus, inferior frontal gyrus, pallidum, inferior parietal lobule, anterior and median cingulate and paracingulate gyri, claustrum, amygdala, medial dorsal nucleus	45, −24, 9	−4.29	Alphasim < 0.05
3	Superior temporal gyrus, middle temporal gyrus, inferior temporal gyrus, precentral gyrus, insula, inferior frontal gyrus, postcentral	45, 6, −18	−4.70	Alphasim < 0.05
4	Precentral gyrus, superior frontal gyrus, middle frontal gyrus, postcentral gyrus, middle frontal gyrus, paracentral lobule	30, 21, 60	−4.96	Alphasim < 0.05
5	Supplementary motor area, superior frontal gyrus, medial frontal gyrus, paracentral lobule	0, −6, 72	−4.80	Alphasim < 0.05

DOC, disorders of consciousness; MNI, Montreal neurological institute.

### Features of functional connectivity between each region of interest pair

**Figure 4:**
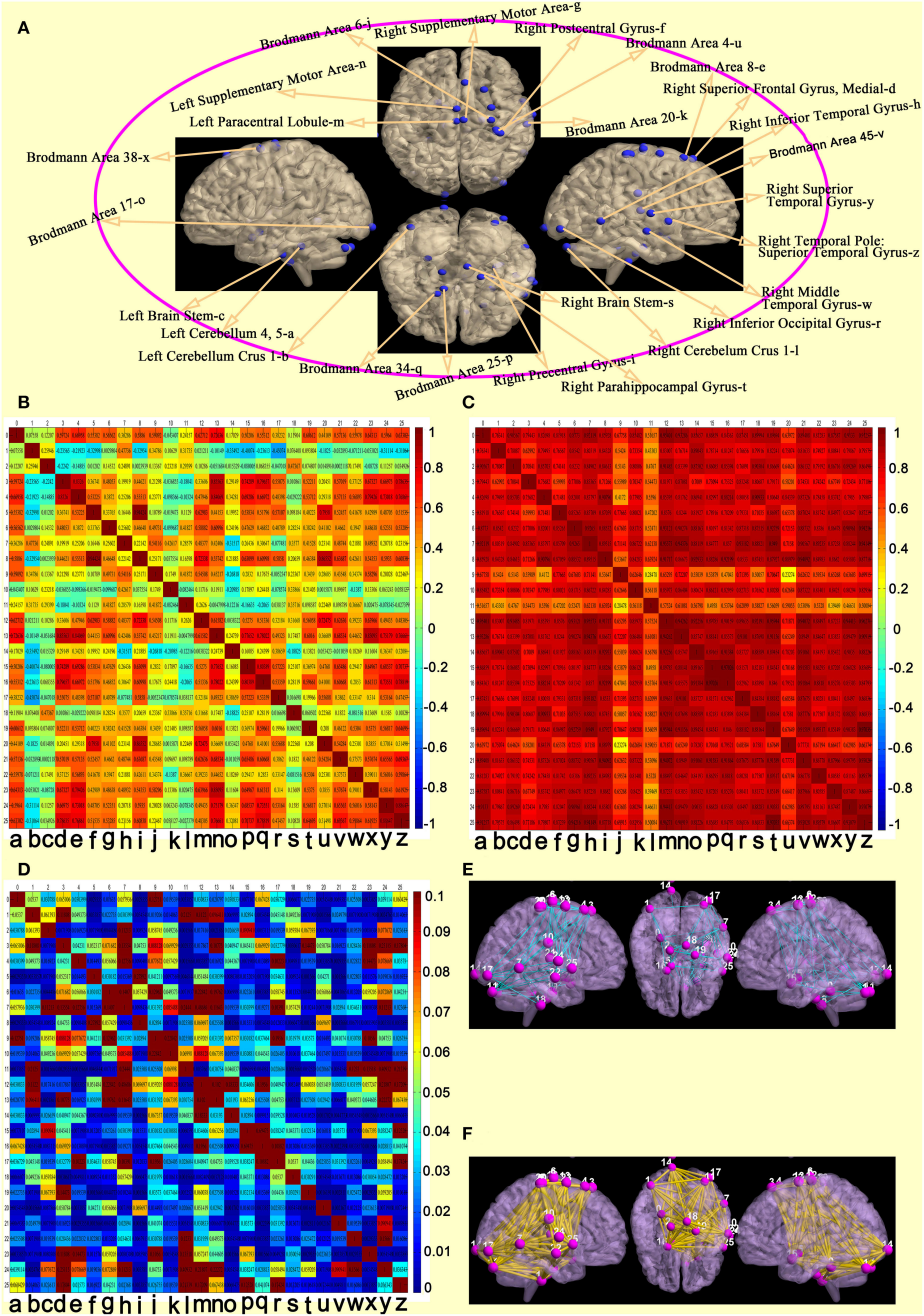
Features of functional connectivity between each regions of interest pair. **(A)** 26 regions of interest. **(B)** matrix of Pearson's correlation coefficients between each regions of interest pair in the awake group. **(C)** matrix of Pearson's correlation coefficients between each regions of interest pair in the DOC group. **(D)** differences of a matrix of *Z*-values between awake and DOC groups. **(E)** compared with the wake group, the strength of functional connectivity between some regions of interest pairs in the DOC group were similar. **(F)** compared with the wake group, the strength of functional connectivity between many regions of interest pairs in the DOC group were enhanced).

The 26 regions of interest were selected, according to the above comparison results of the wake and DOC groups (Figure 4A). Compared with the wake group (Figure 4B), the DOC group (Figure 4C) had a higher Pearson's correlation coefficients (*R*-values) between each region of interest pair. The difference between groups was statistically significant (FDR corrected, *P* < 0.05) (Figure 4D). The visualization findings indicated that, compared with the wake group, the strength of functional connectivity between some regions of interest pairs (28.99%) in the DOC group was similar (Figure 4E), while many regions of interest (71.01%) were enhanced (Figure 4F).

### Quantitative analysis of regions of interest in wake and DOC groups

Z-values of regions of interest were extracted for quantitative comparison between wake and DOC groups. Twenty-six regions of interest were obtained from the comparison results between the wake and DOC groups. The findings of the quantitative analysis are presented in Table 4. Statistically significant differences were found between both groups (*P* < 0.05).

**Table 4:** Quantitative analysis of regions of interests in wake and DOC groups.

**Brain regions**	**Wake group**	**DOC group**	***P*-value**
Left cerebellum 4, 5	1.218 ± 0.346	1.632 ± 0.196	0.006
Left cerebellum crus1	0.473 ± 0.325	0.853 ± 0.449	0.036
Left brain stem	0.758 ± 0.397	1.312 ± 0.507	0.011
Right superior frontal gyrus, medial	0.496 ± 0.346	0.947 ± 0.423	0.015
Brodmann area 8	0.712 ± 0.266	1.371 ± 0.384	<0.001
Right postcentral gyrus	0.769 ± 0.313	1.354 ± 0.365	0.001
Right supplementary motor area	0.384 ± 0.354	0.99 ± 0.477	0.003
Right inferior temporal gyrus	0.737 ± 0.443	1.219 ± 0.340	0.017
Right precentral gyrus	0.782 ± 0.358	1.448 ± 0.358	0.001
Brodmann area 6	0.496 ± 0.345	0.961 ± 0.658	0.046
Brodmann area 20	0.763 ± 0.573	1.379 ± 0.170	0.002
Right cerebelum crus 1	0.318 ± 0.299	0.816 ± 0.272	0.001
Left paracentral lobule	0.860 ± 0.447	1.424 ± 0.339	0.007
Left supplementary motor area	0.680 ± 0.370	1.229 ± 0.355	0.003
Brodmann area 17	0.621 ± 0.335	1.050 ± 0.532	0.034
Brodmann area 25	0.842 ± 0.493	1.311 ± 0.209	0.008
Brodmann area 34	0.934 ± 0.445	1.428 ± 0.235	0.004
Right inferior occipital gyrus	0.561 ± 0.479	1.006 ± 0.461	0.049
Right brain stem	0.627 ± 0.409	1.294 ± 0.408	0.002
Right parahippocampal gyrus;	0.882 ± 0.372	1.441 ± 0.299	0.002
Brodmann area 4	0.553 ± 0.346	1.232 ± 0.422	0.001
Brodmann area 45	1.126 ± 0.426	1.722 ± 0.564	0.013
Right middle temporal gyrus	0.898 ± 0.617	1.565 ± 0.290	0.004
Brodmann area 38	0.569 ± 0.299	1.247 ± 0.435	<0.001
Right superior temporal gyrus	0.976 ± 0.417	1.498 ± 0.445	0.0014
Right temporal pole: superior temporal gyrus	0.972 ± 0.399	1.539 ± 0.516	0.011

DOC, disorders of consciousness.

### The prognostic assessment ability of the indicators

**Figure 5:**
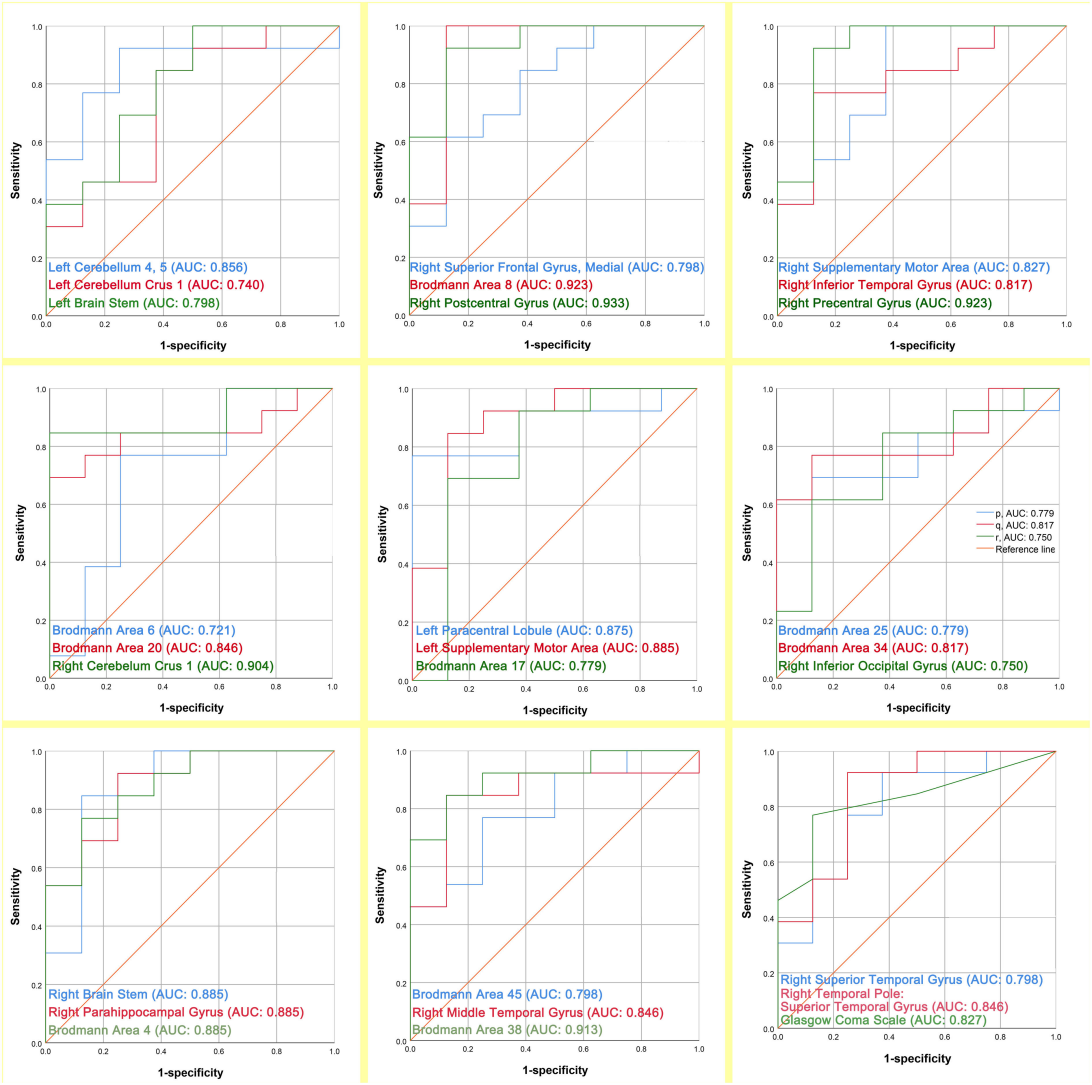
The prognostic assessment ability of the 26 regions of interest and GCS respectively for the outcome of DOC patients (AUC: area under the curve).

The ROC curve analysis was applied to assess the prognostic power of 26 regions of interest and GCS by comparing the area under the curve (AUC) for each ROC. The results of AUC are presented in Figure 5. The prognostic discriminative power of the 26 regions of interest fluctuated between 0.721 and 0.933. Among them, the right postcentral gyrus has the strongest prognostic prediction ability for DOC patients (AUC = 0.933) and Brodmann Area 6 has the weakest power to assess the prognosis of patients with DOC (AUC = 0.721). In addition, the performance of GCS in the prognostic assessment of DOC patients was modest (AUC = 0.827).

### Robustness analysis

**Figure 6:**
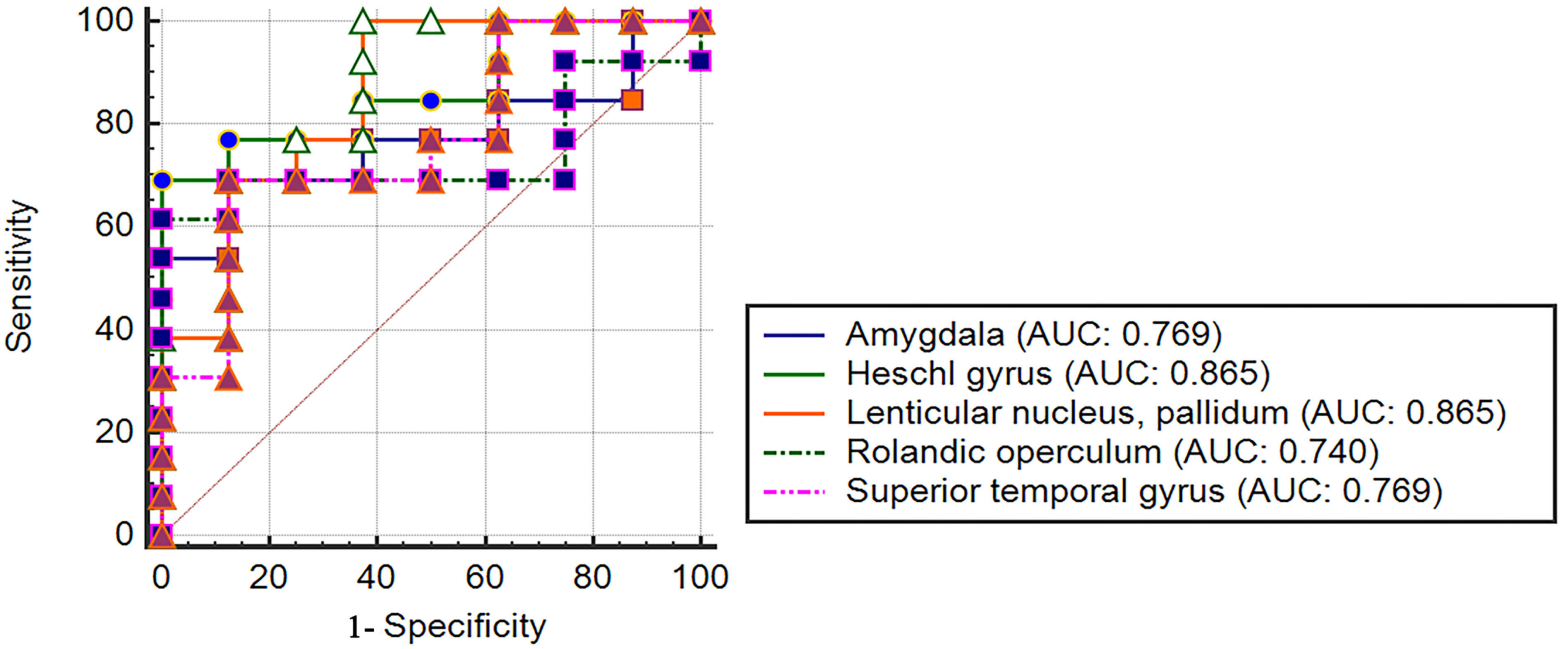
Robustness analysis of prognosis prediction based on AAL template.

The AAL template was used and we aimed to divide the whole-brain into 116 regions of interest. The *Z*-values for functional connectivity of these regions of interest were extracted. Then, the 6-month prognosis of DOC patients was differentiated based on the *Z*-values. The results of the prognostic assessment were performed as for Figure 6. There were five brain regions that could effectively classify (AUC ranging 0.740 from to 0.865) the 6-month prognosis of patients with DOC. The findings are moderately robust (not only do brain regions overlap but also AUC values are similar).

## Discussion

In this study, resting-state brain network findings, based on sTBI patients suffering from DOC, indicated that functional connectivity of the bilateral thalamus to the whole-brain was disrupted in DOC patients. In addition, the functional connectivity between the bilateral thalamus and the whole-brain of the DOC group was different compared with the wake group, and the differences in functional connectivity were associated with prognosis.

Survivors after sTBI may develop DOC, such as vegetative state/unresponsive arousal syndrome or minimally conscious state. Our findings found that the functional connectivity of the thalamus to midbrain, cerebellum, insula, basal ganglia area, frontal lobe, temporal lobe, superior parietal lobule, inferior parietal lobule, limbic lobe, precuneus, cingulate gyrus, paracentral lobule, and occipital lobe was reduced in DOC patients compared with healthy controls. Similar to our results, previous studies showed that thalamic structural integrity and the thalamic-cortical networks were disrupted in DOC (23, 24).

The complex structure of the thalamus determines its functional richness. The thalamus as a relay station plays a critical role in consciousness (23, 24). The thalamus can be divided into a specific projection and a non-specific projection system according to the different types of fiber-endings projecting to the cerebral cortex from various parts of the thalamus and their differences in distribution. The non-specific projection system is closely related to the brainstem reticular system (25). Within the brainstem, neurons of the reticular formation stimulate cortical activation by exciting the widespread projecting neurons of the non-specific thalamocortical projection system (25). Therefore, its main function is to transmit and distribute the upward activation effect originating from the reticular system of the brainstem to almost all cortical areas, maintaining and regulating the excitatory state of the cerebral cortex (influencing the overall level of cortical arousal) (6). Moreover, the formation of consciousness relies on the synergy of multiple parallel systems (6). Once the parallel system is disrupted or interrupted, it may lead to DOC (6, 26).

The cortical network of the consciousness system is very extensive, and mainly includes bilateral cerebral hemispheres, especially the lateral frontal, anterior insula, lateral parietal (adjacent temporo-occipital cortex), medial frontal, medial parietal (precuneus) lobes, anterior lobe, and cingulate cortex (6). Therefore, once trauma involves cortical areas or non-specific projection systems, DOC may appear. Furthermore, due to the devastating violence and widespread spread of violence, the patient's cortical consciousness system was easily disrupted. This may explain the extremely high incidence of DOC in sTBI patients.

Ascending excitatory projections from the thalamus, basal forebrain, and brainstem reticular activating systems (subcortical system) play a serious role in normal cortical excitation during the awake, conscious state (27). The subcortical consciousness system is also not immune to the effects of devastating violence. Evidence from Yu et al. (24) that the microstructural integrity of the thalamus is a critical factor in the generation of consciousness and axonal damage may be the main cause of the disconnection between the thalamus and the cortex. In addition, some additional subcortical structures also play important roles in consciousness and alertness. The superior colliculus and pretectal regions form a core circuit with the pulvinar involved in directed attention (28). The basal ganglia area is reciprocal connections with the thalamic nucleus and this circuit may also be involved in arousal and attention functions (29). In addition, the claustrum nucleus, with extensive cortical connections, has been demonstrated to play a significant role in the maintenance of consciousness and attention (6). The cerebellum is interconnected with the prefrontal cortex to participate in attention and consciousness (30). The results of our study also confirm this, although this finding is presently controversial.

The precuneus and posterior cingulate cortex as the core components of the default mode network are involved in consciousness, introspection, episodic memory, and self-processing (31). A previous study based on diffusion magnetic resonance imaging suggested that the connection between the thalamus and pisteromedial cortex (precuneus and posterior cingulate) white matter cellulose was disrupted in DOC patients (31). This provides a rationale for our findings that structure determines function. However, it should be pointed out that the coupling of structure and function in the field of brain science is not a one-to-one match. Although we are unable to reveal the mechanism of the coupling between brain structure and function, we deeply realize that biophysical and network communication models with optimized structure-function coupling will be more conducive to explain the above findings. Therefore, a scientific and rigorous circuit brain network model of biophysical communication is urgently needed in the DOC field.

Based on the above findings, we further analyzed the network of the thalamus-to-whole-brain in patients with different prognoses (wake group *vs*. DOC group) 6 months after sTBI. As expected, the functional connectivity of the thalamus to the whole-brain was abnormally increased in the DOC group compared with the wake group. This may be related to the disconnection between the thalamus and consciousness system (the cortex and the cortical subconscious) in DOC group patients. On the surface, the functional connectivity of patients with DOC is enhanced and in essence, there is no causal relationship between the both, but the local brain activity of compensatory synchronicity self-enhanced between the brain regions after the loss of connectivity (32). Disruption of long-range structural connection (white matter cellulose or neurotransmitter conduction) of the thalamus to the whole-brain is considered a plausible explanation in the acute phase of DOC (32). To clarify the above theory, we manually divided 26 regions of interest based on brain regions with peak differences between wake and DOC groups, and performed functional connectivity analysis of local and distant brain areas. The results showed that most of the adjacent brain regions had enhanced functional connectivity. Although this is an exploratory analysis, it further clarifies our findings. Therefore, it is necessary to further perform long-range causal connectivity and white matter cellulose tracing analysis about acute patients with sTBI in future studies.

Functional connectivity between the thalamus and the whole-brain may be applied as an imaging marker for the prognosis assessment of DOC patients, and it could also be used as an evaluation index for the remaining brain network about latent consciousness function. Differences in neurological outcomes (wake or persistent DOC) in patients with DOC may be associated with their residual brain networks of consciousness systems. Therefore, we cautiously believe that protecting and remodeling the remaining brain consciousness network is the key to improving the wake. Furthermore, the 26 regions of interest defined according to the functional connectivity differences (wake group *vs*. DOC group) had a similar or even better wake representation ability than the admission GCS and a supplementary analysis using an AAL also demonstrated the robustness of the results of this study. Notably, brainstem regions were not included in the AAL template. Thus, a core template based on brainstem areas is urgently needed in the DOC field, although this is moderately challenging. The GCS score is a standardly used measure of neurological status and prognosis (33). However, GCS scores are relatively subjective and are greatly affected by external factors. If the GCS is complemented with functional connectivity (relatively objective and effectively reflect brain activity), their prognostic assessment ability may be more stable and reliable. Based on this protocol in future studies, it is potentially feasible to introduce machine learning to identify the prognosis of sTBI-type DOC patients.

However, our study had several shortcomings. First, the sample size in this study was small. Therefore, machine learning and linear model for prognostic assessment are relatively constrained. Second, DOC is just a general term for the vegetative state, unresponsive arousal syndrome, minimally conscious state, etc. This study failed to further differentiate and compare them. Third, the age mismatch between healthy controls and DOC patients, although adjusted for its effect, may also have biased the results. Fourth, coupling the structural and functional networks is an ideal protocol for in-depth exploration of DOC mechanisms and performing prognostic assessments. However, limited to the study protocol, we failed to make further explorations.

## Conclusion

The thalamus-based system of consciousness of sTBI patients suffering from DOC is disrupted. There are differences in the thalamus-to-whole-brain network between wake and DOC groups. These differences may set the tone for the direction of neurological outcomes in patients and have potential prognostic characterization capability. Future studies with larger sample sizes, ideal structure-function coupling protocol, and machine learning model are needed to clarify mechanisms and prognostic classification ability.

## Data availability statement

The raw data supporting the conclusions of this article will be made available by the authors, without undue reservation.

## Ethics statement

The studies involving human participants were reviewed and approved by Ethics Committee of Clinical Medical College of Yangzhou University (2020KY-179). The patients/participants provided their written informed consent to participate in this study.

## Author contributions

Conception and design: LD, JZ, and XiaW. Data collection: JZ, LD, and HYZ. Data analysis: JZ. Drafting: JZ, HZZ, and FLY. Helping with drafting: EPZ, ZJY, and YPL. Draft revision: YLP, MW, XinW, and XiaW. All authors contributed to the article and approved the submitted version.

## Funding

This study was funded by the Jiangsu Province High-level Health Talents Six Ones Project Top-notch Talent Research Project (LGY2018031) and the Hospital Level Support Projects (Fcjs202050).

## Conflict of interest

The authors declare that the research was conducted in the absence of any commercial or financial relationships that could be construed as a potential conflict of interest.

## Publisher's note

All claims expressed in this article are solely those of the authors and do not necessarily represent those of their affiliated organizations, or those of the publisher, the editors and the reviewers. Any product that may be evaluated in this article, or claim that may be made by its manufacturer, is not guaranteed or endorsed by the publisher.

## Abbreviations

sTBI: Severe traumatic brain injury
DOC: Disorders of consciousness
fMRI: Functional magnetic resonance imaging
GCS: Glasgow coma score
MRI: Magnetic resonance imaging
GOS-E: Extended Glasgow Outcome Score
AAL: Automated anatomical labeling
FDR: False discovery rate
ROC: Receiver operating characteristic
AUC: Area under the curve.
